# Flow cytometry data analysis of CD34+/CD133+ stem cells in bone marrow and peripheral blood and T, B, and NK cells after hematopoietic grafting

**DOI:** 10.1016/j.dib.2016.03.078

**Published:** 2016-03-31

**Authors:** José C. Jaime-Pérez, César D. Villarreal-Villarreal, Eduardo Vázquez-Garza, Nereida Méndez-Ramírez, Rosario Salazar-Riojas, David Gómez-Almaguer

**Affiliations:** Department of Hematology, Internal Medicine Division, “Dr. José E. González” University Hospital of the School of Medicine of the Universidad Autónoma de Nuevo León, Monterrey, México

**Keywords:** Bone Marrow, CD34+ cells, CD133+ hematoprogenitors, Flow cytometry, Hematopoietic transplant, Peripheral blood, T, B and NK cells

## Abstract

This article provides flow cytometry information regarding levels of expression for hematopoietic stem cell markers CD34 and CD133 obtained simultaneously of the bone marrow and peripheral blood from recipients of allogeneic and autologous transplants of PB hematoprogenitors for treating hematological malignancies and who were clinically healthy after ≥100 days following the procedure. CD34 and CD133 expression is compared regarding type of transplant (autologous vs. allogeneic) and sample cell source (bone marrow vs. peripheral blood). Patients were conditioned with a reduced-intensity conditioning regimen. Also shown is the flow cytometry analysis of mononuclear cell and lymphocyte populations in the peripheral blood of both types of recipients, as well as the characterization of immune cells, including T lymphocyte antigenic make up markers CD3, CD4 and CD8, B lymphocytes and NK cells, including total NK, bright and dim subtypes in the peripheral blood of both types of recipients. For further information and discussion regarding interpretation and meaning of post-transplant flow cytometry analysis, please refer to the article “Assessment of immune reconstitution status in recipients of a successful hematopoietic stem cell transplant from peripheral blood after reduced intensity conditioning” [1].

**Specifications table**

**Table:** 

Subject area	Immunology
More specific subject area	Transplantation hematology
Type of data	Figures
How data was acquired	Flow cytometry
Data format	Analyzed
Experimental factors	Mononuclear cells were suspended in PBS with bovine serum albumin and sodium azide in round-bottom tubes and incubated in the dark.
Experimental features	Hematoprogenitor and immune mononuclear cells were stained with pertinent fluorochrome-marked antibodies, then analyzed by flow cytometry.
Data source location	Universidad Autónoma de Nuevo León, Monterrey, México
Data accessibility	*Data is with this article.*

## Value of the data

• Flow cytometry data of bone marrow and peripheral blood of hematology patients who received a stem cell transplant provides a precise assessment of immune status after grafting is complete.
• This is the first report of the correlation between CD34+ and CD133+ hematoprogenitors in bone marrow and peripheral blood after hematopoietic grafting.
• The data provide an experimental guide to further define post-transplant reconstitution dynamics in humans through correlative studies.

## 1 Data

**Fig. 1:**
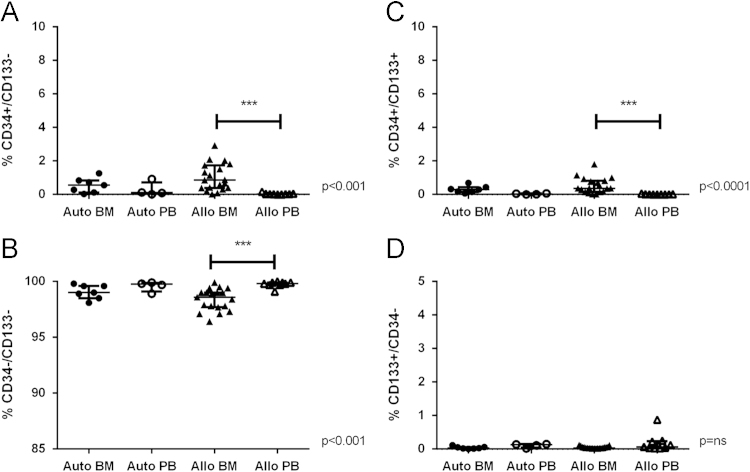
A. CD34+/CD133−, B**.** CD34−/CD133−, and C**.** CD133+/CD34+cells in the bone marrow compartment from allogeneic (Allo) transplant recipients compared to the rest of the compartments and transplants (*p*<0.001, *p*<0.0001 and *p*<0.001 respectively). D**.** Values of CD133+/CD34− cells were similar among groups with no statistical significance. Kruskal–Wallis test was used to compare the groups, with Tukeys post-hoc test^⁎⁎⁎^*p*<0.001.

**Fig. 2:**
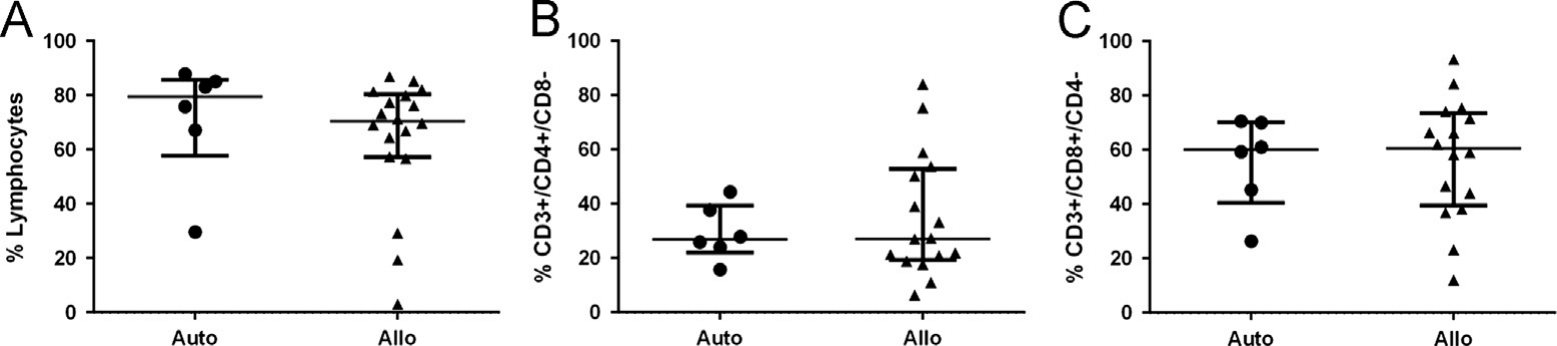
The main populations analyzed were A. Total lymphocytes, B. CD4+T-lymphocytes and C. CD8+T-lymphocytes.

**Fig. 3:**
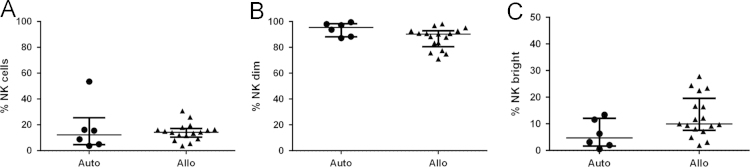
A. Natural Killer (NK) cells with their respective subsets B. CD56-dim and C. CD56-bright.

**Fig. 4:**
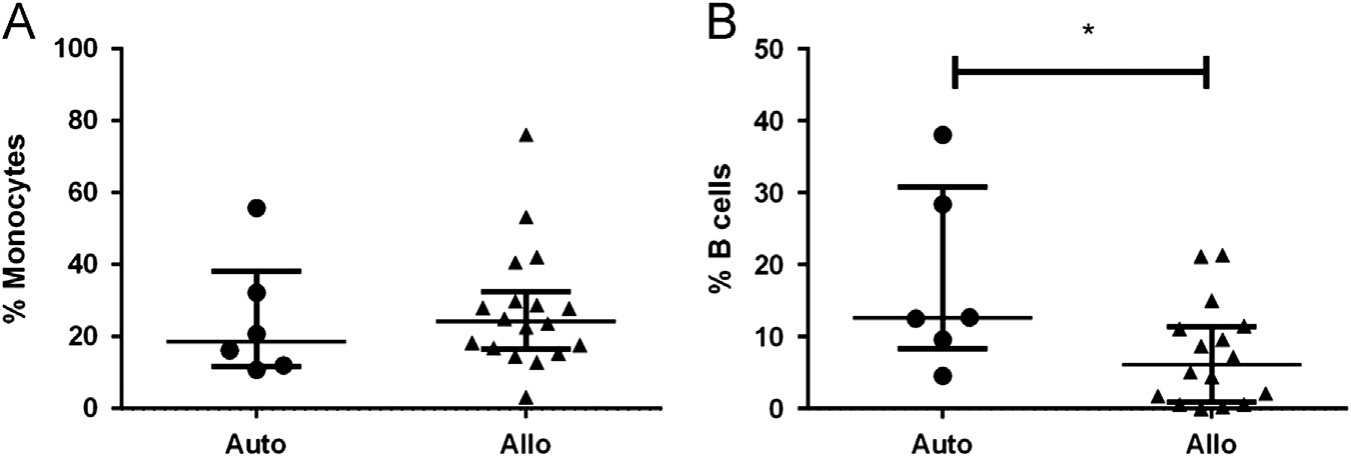
A. Monocytes and B**.** B**-**lymphocytes, (Mann U Whitney test was performed for comparisons, ^⁎^*p*<0.05).

Here, we exemplify the application of CD34/CD133 antigenic make-up analysis in mononuclear cells after hematopoietic stem cell transplantation (HSCT) by flow cytometry, comparing between type of transplant, autologous (Auto) vs. allogeneic (Allo), and cell source, bone marrow (BM) vs. peripheral blood (PB). Figure panels depicting the median and interquartile ranges (25,75) of the percentage values of different hematoprogenitor populations expressing the characteristic markers CD34 and CD133 are shown (Fig. 1). To assess their individual contribution to immune cell composition in PB according to transplant type, T (Fig. 2) NK (Fig. 3) and B (Fig. 4) lymphocytes were studied. Data depicting the median values and interquartile ranges are presented, comparing the ratio of the different cell components.

## 2 Experimental design, materials and methods

### 2.1 Cell count and sample preparation for staining

To analyze the data of each peripheral blood sample, a complete blood count was performed with a Sysmex XS-1000i (Lincolnshire, IL, USA). For antibody staining, 1×10^6^ WBC were suspended in a total volume of 100 μL containing phosphate-buffered saline (PBS), (Miltenyi Biotec) with 0.5% BSA (bovine serum albumin) and 0.09% sodium azide (BD Biosciences) in BD Falcon round-bottom tubes (BD Biosciences).

### 2.2 Antibody panel

We consulted several panels used in relevant literature for sample acquisition [2], flow cytometry panels in heterogeneous blood samples [3], [4], [5] and recent publications of hematoprogenitor cells [6], [7], [8]. For hematoprogenitor characterization assays in BM and PB, anti-CD34 APC (clone 8G12, BD Biosciences) and anti-CD133 PE (clone 293C3, Miltenyi Biotechnology) were used. To identify mononuclear immune cells in PB the following panel was employed: anti-CD3 PerCP (clone UCHT1, DAKO), anti-CD4 V450 (clone RPA-T4, BD Biosciences), anti-CD8 FITC (clone SK1, BD Biosciences), anti-CD19 APC-H7 (clone SJ25-C1, BD Biosciences), anti-CD45-V500 (clone 2D1, BD Biosciences), anti-CD45 FITC (clone 2D1, BD Biosciences) and anti-CD56 PE (clone MY31, BD Biosciences). After staining, samples were incubated for 15 min in the dark.

### 2.3 RBC lysing and sample preparation before FACS analysis

The samples were prepared as previously described [9]. After incubation cells were suspended with 2.0 mL of a 1/10 dilution FACSLysing solution (BD Biosciences) and incubated in the dark for 10 min at room temperature. Cells were centrifuged at 540 gx5 min, the supernatant was discarded using Pasteur pipettes and the cell pellet suspended in 50 μL of buffer solution. Cells were then washed with 2.0 mL of PBS containing 0.5% BSA and 0.09% sodium azide, vortexed, and centrifuged at 540 g x 5 min. Finally, the supernatant was discarded and the cells suspended in 200 μL of PBS containing 0.5% BSA. Cells were kept at 4 °C before analysis.

### 2.4 Determination of population by flow cytometry analysis

Dead cells and debris were discarded by using Forward scatter/Side scatter (FSc/SSc) exclusion in the sample dotplots; the main location of lymphocytes and monocytes was considered for the gating strategy. Doublets were excluded by double forward (FSC-A and FSC-H) and side scatter (SSC-A and SSC-H), prior to gating the relevant sub populations [10]. The methodology to gate cell percentages was based on previous experience in the laboratory following relevant references regarding hematoprogenitor cell [11], and mononuclear [12], [13], [14] cell gating. The populations were deemed as follows: Hematoprogenitors as CD34+, CD133+or CD34+/CD133+ (Fig. 1). Lymphocytes and monocytes being CD45+. Granulocytes were excluded by size and location in SSc vs. CD45. Total T cells being the CD45+/ CD3+population; from these cells the proportions of CD4 and CD8+cells were determined (Fig. 2). NK cells were deemed as CD45+/ CD3-/CD56+, their dim and bright subpopulations were gated according to the density of expression of CD56 (Fig. 3). B-Lymphocytes were analyzed through CD3−/CD56−/CD19+expression (Fig. 4).

Blank samples and FMOs (Fluorescence minus one) were used to determine the location of the gating in both, mononuclear and hematoprogenitor cell locations. FMO controls consist of all staining except one, and is used to determine proper electronic gating [15], [16]. All samples were analyzed in a FACSCanto II flow cytometer (BD Biosciences). The data was collected and analyzed using SPSS v.20 and Prism software.

## Conflicts of interest

None. The authors confirm that there are no conflicts of interest in the present submission.
